# Dietary advanced glycation end-product consumption leads to mechanical stiffening of murine intervertebral discs

**DOI:** 10.1242/dmm.036012

**Published:** 2018-12-18

**Authors:** Divya Krishnamoorthy, Robert C. Hoy, Devorah M. Natelson, Olivia M. Torre, Damien M. Laudier, James C. Iatridis, Svenja Illien-Jünger

**Affiliations:** Leni & Peter W. May Department of Orthopaedics, Icahn School of Medicine at Mount Sinai, New York, NY 10029, USA

**Keywords:** Spine, Intervertebral disc degeneration, Diabetes, Advanced glycation end-products, Diet

## Abstract

Back pain is a leading cause of disability and is strongly associated with intervertebral disc (IVD) degeneration. Reducing structural disruption and catabolism in IVD degeneration remains an important clinical challenge. Pro-oxidant and structure-modifying advanced glycation end-products (AGEs) contribute to obesity and diabetes, which are associated with increased back pain, and accumulate in tissues due to hyperglycemia or ingestion of foods processed at high heat. Collagen-rich IVDs are particularly susceptible to AGE accumulation due to their slow metabolic rates, yet it is unclear whether dietary AGEs can cross the endplates to accumulate in IVDs. A dietary mouse model was used to test the hypothesis that chronic consumption of high AGE diets results in sex-specific IVD structural disruption and functional changes. High AGE diet resulted in AGE accumulation in IVDs and increased IVD compressive stiffness, torque range and failure torque, particularly for females. These biomechanical changes were likely caused by significantly increased AGE crosslinking in the annulus fibrosus, measured by multiphoton imaging. Increased collagen damage measured with collagen hybridizing peptide did not appear to influence biomechanical properties and may be a risk factor as these animals age. The greater influence of high AGE diet on females is an important area of future investigation that may involve AGE receptors known to interact with estrogen. We conclude that high AGE diets can be a source for IVD crosslinking and collagen damage known to be important in IVD degeneration. Dietary modifications and interventions that reduce AGEs warrant further investigation and may be particularly important for diabetics, in whom AGEs accumulate more rapidly.

## Abstract (precis)

**Summary:** Dietary AGEs lead to sex-specific intervertebral disc structural and functional changes and may be targeted for promoting spinal health, especially in diabetes, in which AGEs form rapidly.

## INTRODUCTION

Intervertebral disc (IVD) degeneration is a progressive condition and major contributor to back pain, which is a leading cause of global disability and absence from work (Hartvigsen et al., 2018; Adams and Roughley, 2006). Pain from IVD degeneration arises from accumulation of structural disruption, catabolism and chronic inflammatory conditions in the IVD. Consequently, it is a clinical priority to identify ways to reduce IVD damage. Risk factors for IVD degeneration are multifactorial, and recent studies have shown that diabetes mellitus (DM) and obesity (or being overweight) add significant morbidity to back pain and IVD degeneration (Liuke et al., 2005; Takatalo et al., 2013; Jakoi et al., 2017; Samartzis et al., 2013; Fabiane et al., 2016) as they increase the risk for herniation (Sakellaridis, 2006), spinal stenosis (Anekstein et al., 2010) and spinal surgery complications (Guzman et al., 2014). DM and obesity are increasing at alarming rates, making this a growing high-risk population. Furthermore, DM and obesity contribute to chronic inflammation, catabolism and altered biomechanics on the spine (Fields et al., 2015; Hillson, 2018; Illien-Jünger et al., 2013), and we believe that understanding risk factors for DM and obesity will also help elucidate novel risk factors for spinal diseases in the general population. Interestingly, sex differences in obesity and DM prevalence exist, with DM women having increased risks for cardiovascular disease, myocardial infarction and stroke mortality compared to DM men (Kautzky-Willer et al., 2016). The existence of sex-dimorphic pathologies in DM and other diseases motivates further investigation of sex differences in spinal diseases.

Advanced glycation end-product (AGE) accumulation is a source for DM complications, and known to increase the risk for arthrosclerosis (Saremi et al., 2017), retinopathy and renal failure (Beisswenger et al., 1995). AGEs also accumulate to a greater degree in diabetic human IVDs, where they are associated with increased matrix-degrading enzymes (Tsai et al., 2014). In rats, type II DM was associated with degenerative changes in IVDs, including glycosaminoglycan loss and IVD stiffening, which were again attributed to AGE accumulation (Fields et al., 2015). We previously demonstrated that type I DM mice had increased IVD structural disruption and pro-inflammatory cytokines that were associated with AGE accumulation (Illien-Jünger et al., 2013). We believe that AGEs are a likely source of crosslinking and catabolism in the IVD, yet it is not clear whether AGEs can accumulate in the avascular IVD from ingestion of high AGE diets or require hyperglycemic conditions from DM.

AGEs are highly oxidant compounds that can accumulate in tissues through endogenous (i.e. hyperglycemia) and exogenous (i.e. thermally processed foods) sources; 10% of dietary AGEs are absorbed via the intestine and released into the bloodstream (Koschinsky et al., 1997). Alarmingly, over the past 20 years, consumption of western diets consisting of highly processed foods has significantly increased, also increasing the prevalence of obesity and DM (Cordain et al., 2005).

Recently, we showed in aging pre-diabetic mice that chronic ingestion of diets enriched with the specific AGE precursor (methylglyoxal) accelerated age-related vertebral bone loss and induced AGE accumulation within the endplate (Illien-Jünger et al., 2015). When further investigated, ingestion of dietary AGEs induced sex-dependent bone loss with inferior biomechanical properties in young (6 months) female mice (Illien-Jünger et al., 2018). These studies provided the first evidence that dietary AGEs had a direct effect on vertebral structure and function, and also showed that this effect was sex dependent. Furthermore, although the endplate had strong immunostaining for AGEs, it remains unclear whether dietary AGEs can accumulate in IVDs and contribute to structural or catabolic changes known to be present in degeneration. Therefore, the aim of the current study was to assess the effects of dietary AGEs on structure and function of IVDs in female and male mice. We hypothesize that chronic ingestion of a high AGE diet will accumulate in IVDs and result in sex-specific IVD structural disruption and functional changes involving increased AGE crosslinking and collagen damage.

## RESULTS

### General observation

While there were no significant differences between low (L)-AGE and high (H)-AGE female mice [body weights: L-AGE=26.6±4.1 g, H-AGE=26.3±2.6 g, nonsignificant (n.s.); fasting blood glucose: L-AGE=79.2±15.3 mg/dl, H-AGE=84.2±18.7 mg/dl, n.s.], male H-AGE mice had slightly decreased body weight and increased fasting blood glucose compared to male L-AGE mice (body weights: L-AGE=29.6±1.2 g, H-AGE=27.3±1.3 g, *P*=0.002; fasting blood glucose: L-AGE=71.2±10.6 mg/dl, H-AGE=88.4±11.6 mg/dl, *P*=0.006). Food consumption, recorded during the last month prior to sacrifice, indicated significantly lower chow intake in H-AGE compared to L-AGE mice of both sexes (female: L-AGE=4.6±0.4 g/day, H-AGE=3.1±0.3 g/day, *P*<0.001; male: L-AGE=5.0±0.4 g/day, H-AGE=3.0±0.1 g/day, *P*<0.001). However, these immense differences (1 g/day) between diet consumption were likely due to chow loss due to the crumbly consistency of the L-AGE diet, rather than to an actual decrease in chow consumption in H-AGE mice. Importantly, no difference in chow consumption was observed between sexes within the same diet.

### Dietary AGEs led to AGE accumulation in female IVDs

**Fig. 1.**
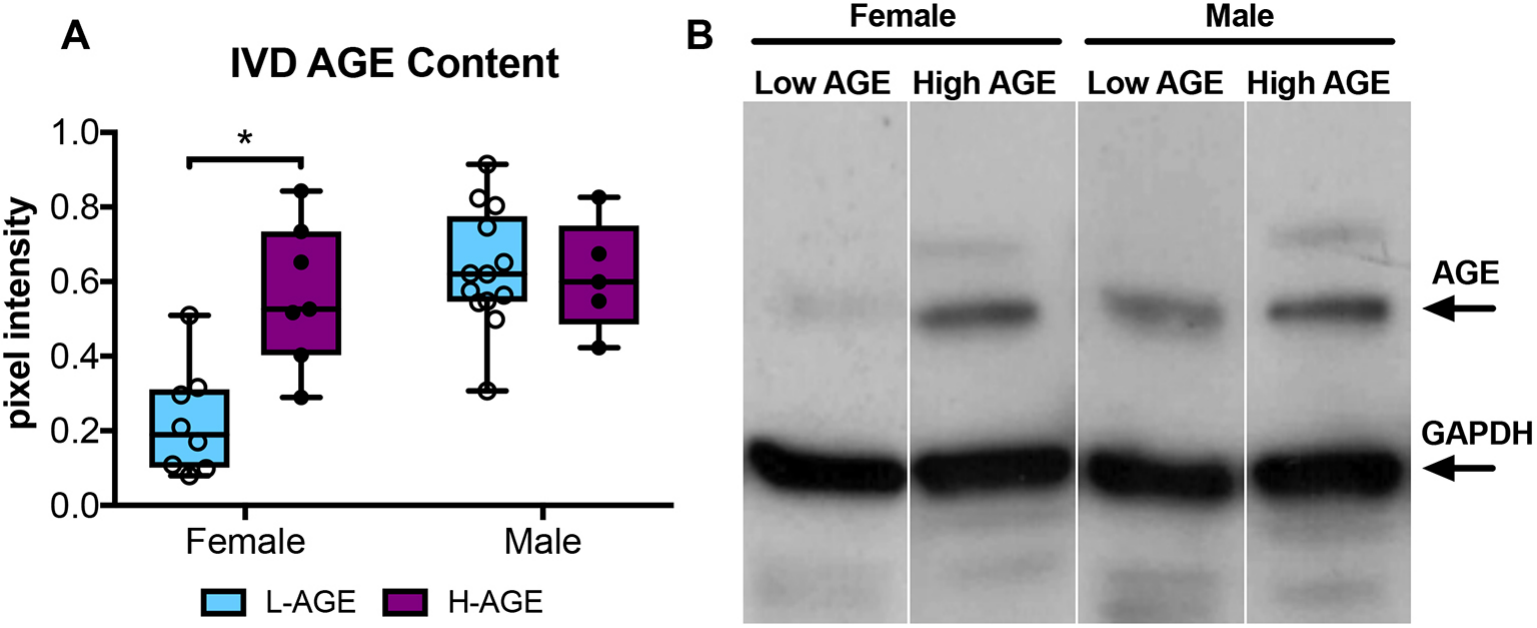
**AGE accumulation in the IVD.** (A) Western blot analysis showed greater AGE protein content in H-AGE female (*n*= 7) IVDs compared to L-AGE females (*n*=9), with no differences between H-AGE (*n*=5) and L-AGE (*n*=13) males. Data are presented as box plots from minimum to maximum±s.d. *P*-values are based on two-tailed unpaired Student's *t*-test with Bonferroni correction and significant if *P*≤0.05 (*). (B) Representative AGE western blot with representative bands for AGE and GAPDH (internal control). AGE-BSA positive control was used to validate the measured AGE bands. Due to low protein concentration within some specimens, some samples needed to be pooled to warrant correct measurements.

H-AGE diet led to significant accumulation of total AGEs in IVDs (*P*=0.003, Fig. 1) of female H-AGE compared to female L-AGE mice as seen by western blot analysis. While multiple AGEs are expected in serum and IVDs, the western blot data revealed the most prominent band at approximately 70 kDa, which is the estimated size for carboxymethyl lysine, one of the most abundant and prevalent AGEs found *in vivo* (Ikeda et al., 1996; Schleicher et al., 1997). We believe that carboxymethyl lysine was the primary AGE being detected through this western blot method. The increase in AGEs is in line with the observed increase in circulating total AGEs found in H-AGE compared to L-AGE females as seen through serum ELISA analysis (16.9±4.3 U/ml vs 9.5±4.8 U/ml, *P*=0.01). No differences in AGE levels in IVDs or serum were observed for male mice (serum AGE, male: 11.7±4.2 U/ml (H-AGE) vs 8.3±.1.6 U/ml  (L-AGE), n.s.). These data suggest that dietary AGEs accumulate systemically and likely also accumulate within IVD tissue in the absence of DM or obesity in female mice. The dietary effect of AGE accumulation is further supported by our previous study, in which H-AGE diet increased serum AGE content by 80%, whereas aging resulted only in 60% serum AGE accumulation in female mice (Illien-Jünger et al., 2018). No differences were observed in male mice.

### Dietary AGEs altered biomechanical properties of female IVDs

**Fig. 2.**
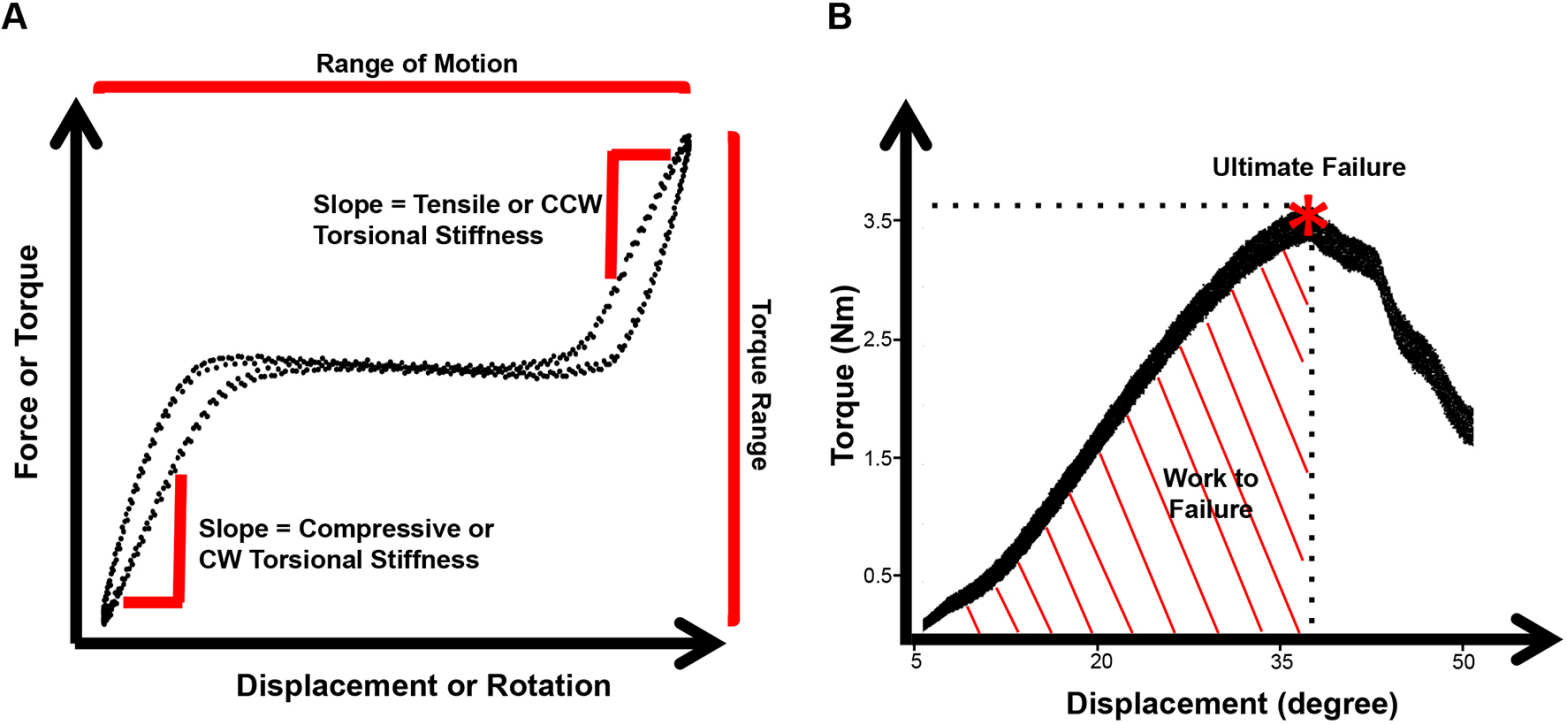
**Biomechanical testing analyses.** (A) Schematic of force-displacement curves for axial tension-compression and torsional testing showing linear regions used for stiffness measurements, regions measured for axial range of motion and torque range. CW, clockwise; CCW, counter-clockwise. (B) Torsion to failure curve schematic showing the point of ultimate failure and area under curve calculated as work done to failure.

**Fig. 3.**
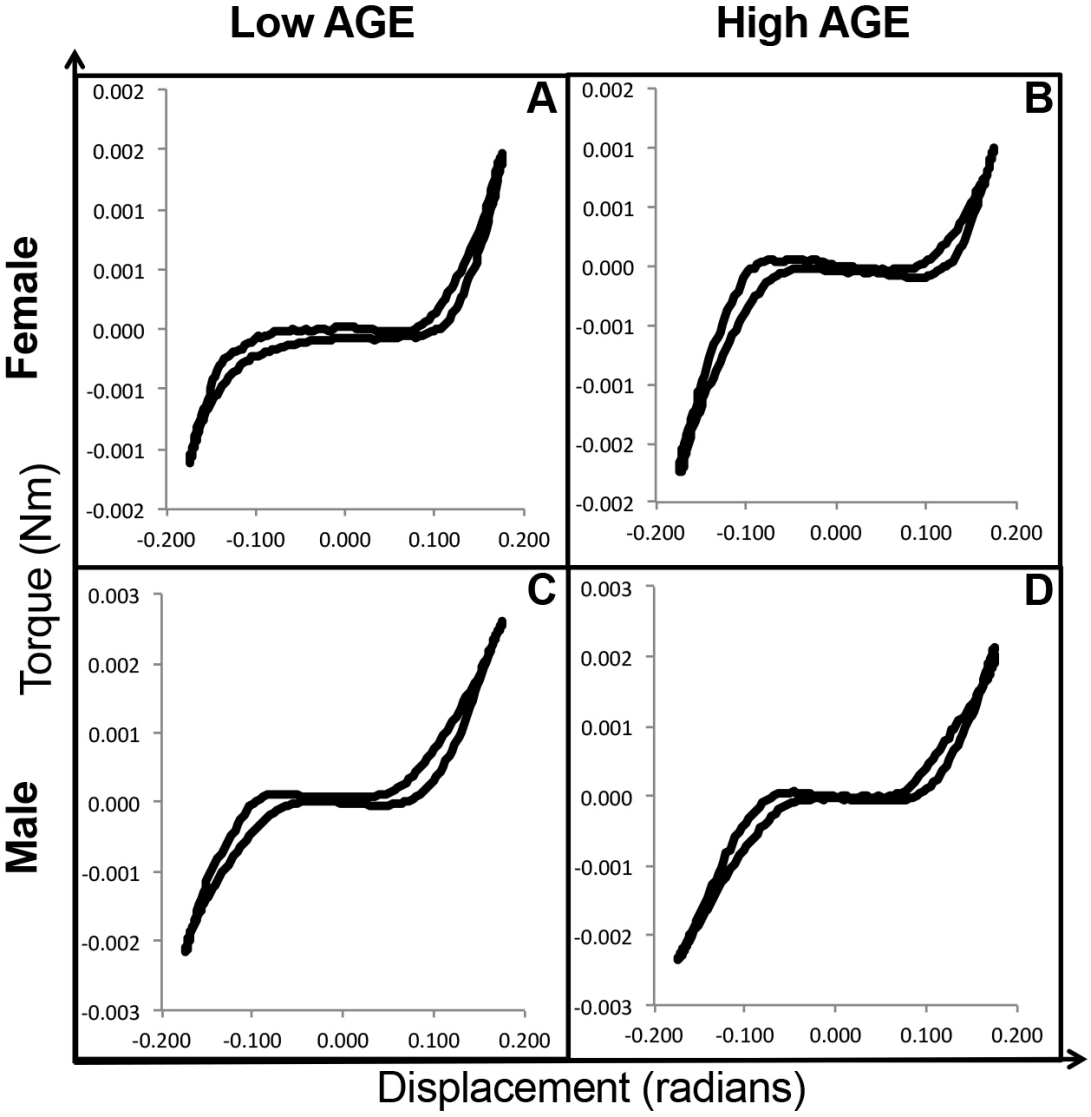
**Representative torque-displacement curves.** These curves represent the second to last cycle of torsion loading of (A,B) female and (C,D) male L-AGE and H-AGE motion segments, respectively.

**Fig. 4.**
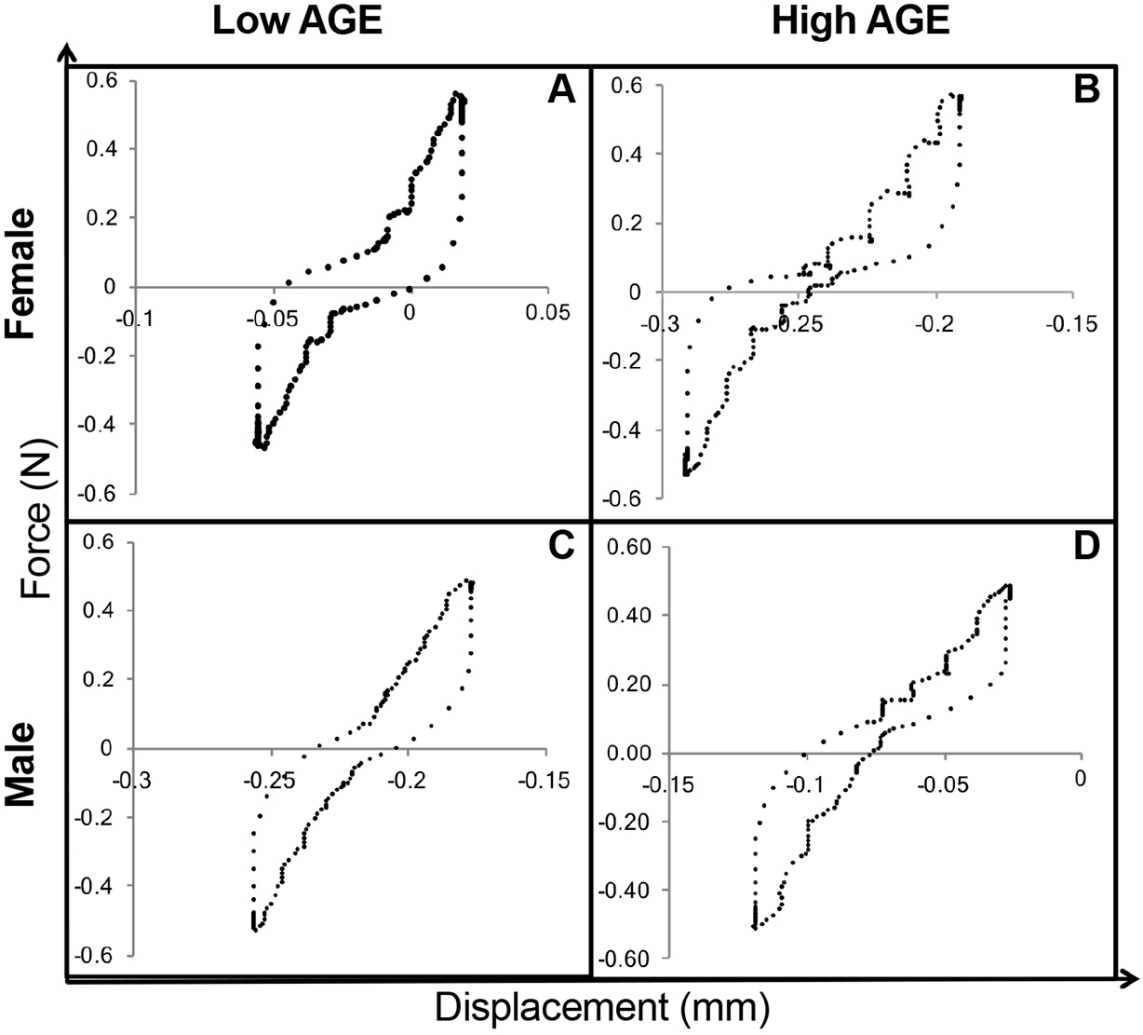
**Representative force-displacement curves.** These curves represent the second to last cycle of axial tension-compression loading of (A,B) female and (C,D) male L-AGE and H-AGE motion segments, respectively.

**Fig. 5.**
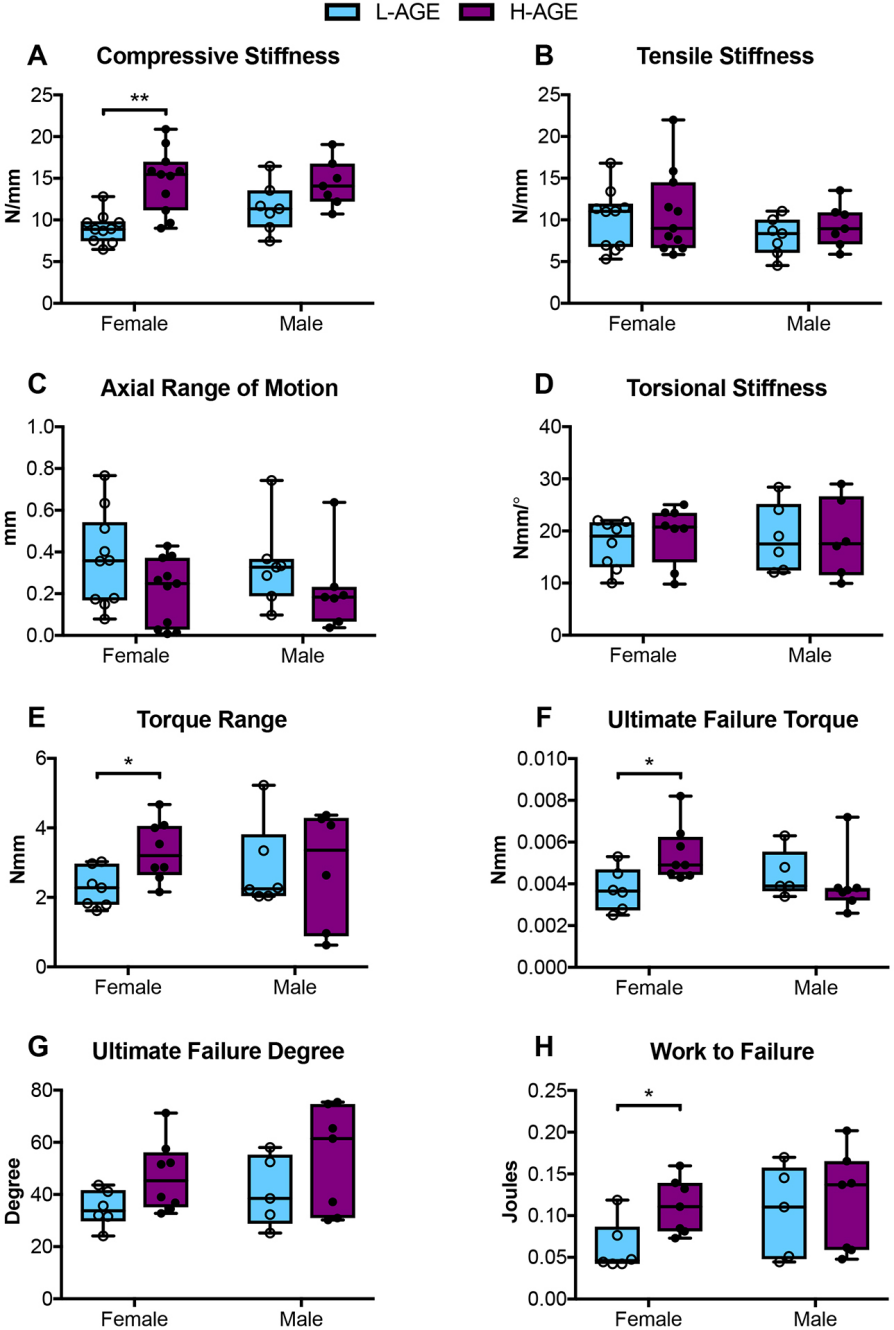
**Tension-compression and torsion biomechanical testing.** (A) Compressive stiffness increased in H-AGE female (‘F’) (*n*=11) compared to L-AGE F (*n*=10), with no differences in (B) tensile stiffness, (C) axial range of motion or (D) torsional stiffness. (E) Torque range was increased in H-AGE F (*n*=8) compared to L-AGE F (*n*=7). Failure analysis revealed that (F) ultimate failure torque and (H) work to failure increased in H-AGE F (*n*=7) compared to L-AGE F (*n*=6), with no difference in the (G) ultimate failure degree. No differences were detected between L-AGE males (‘M’) (*n*=5) and H-AGE M (*n*=7). Data are presented as box plots from minimum to maximum±s.d. *P*-values are based on two-tailed unpaired Student's *t*-test with Bonferroni correction and significant if *P*≤0.05. *P≤0.05, **P≤0.001.

Functional assessment of IVDs was performed by axial compression-tension and torsion biomechanical testing on caudal IVD motion segments. Biomechanical properties, as shown in Fig. 2, were calculated from the second to last cycle of the torque-rotation (Fig. 3) and force-displacement (Fig. 4) curves. IVDs of female H-AGE mice had significantly increased compressive stiffness (+70%, *P*<0.001, Fig. 5A) and increased torque range (+47%, *P*=0.031, Fig. 5E) compared to female L-AGE mice. Failure analysis indicated that ultimate failure occurred at a greater torque in female H-AGE compared to L-AGE mice (+46%, *P*=0.05, Fig. 5F) and, similarly, work done to failure, which is comparable to material toughness, was also increased in female H-AGE compared to L-AGE mice (+129%, *P*=0.04, Fig. 5H). No differences were observed for tensile stiffness, axial range of motion, torsional stiffness (Fig. 5B-D) or IVD cross-sectional areas (cross-sectional area: L-AGE: 1.89±0.24 mm^2^; H-AGE: 1.75±0.28 mm^2^, *P*>0.05). Male IVDs were not affected by diet in any of the measured biomechanical parameters (Fig. 5; cross-sectional area: L-AGE=1.65±0.39 mm^2^, H-AGE=1.78±0.17 mm^2^, *P*>0.05). These data show that motion segment behavior in compression and torsion is altered in female mice on H-AGE diet.

### Dietary AGEs altered annulus fibrosus organization in female IVDs

**Fig. 6.**
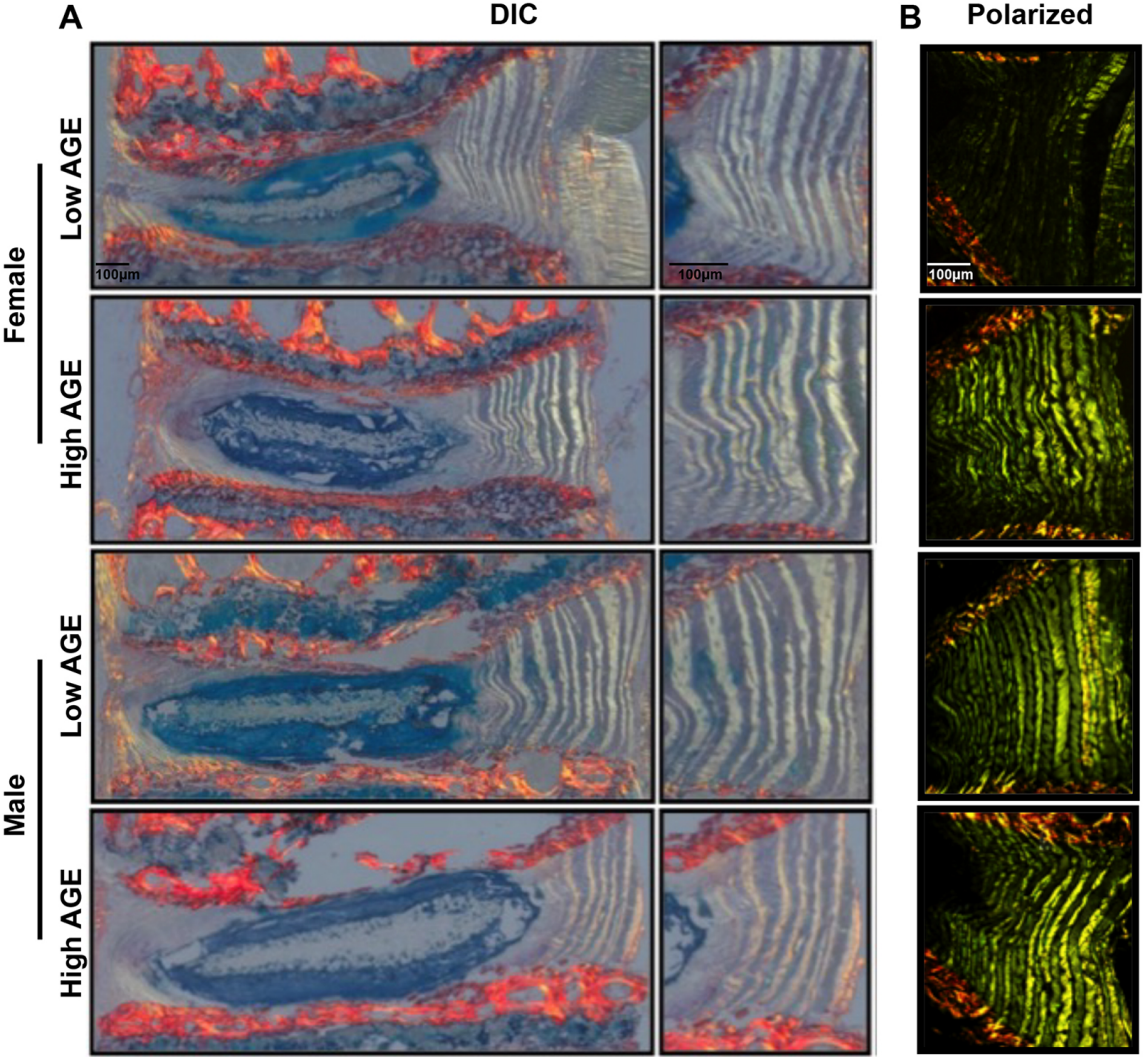
**Histological analysis of IVD morphology.** Representative sagittal sections stained with Picrosirius Red/Alcian Blue (PR/AB) imaged under (A) differential interference contrast (DIC) and (B) polarized light. No differences were observed in end plate and nucleus pulposus between females of either diet. Polarized images showed a difference in the brightness of annulus fibrosus (AF) collagen fibers between the L-AGE (*n*=5) and H-AGE (*n*=8) females. No differences were observed between L-AGE (*n*=5) and H-AGE (*n*=7) males. Scale bars: 100 μm.

To assess morphological changes in the IVD, Picrosirius Red and Alcian Blue (PR/AB)-stained sections were imaged under differential interference contrast (DIC) and polarized filters. DIC imaging revealed no major changes in morphology in the nucleus pulposus or endplate (Fig. 6A). However, when imaged under polarized filters, annulus fibrosus (AF) collagen fibers of female mice appeared brighter in the H-AGE compared to L-AGE group, with no differences between male IVDs (Fig. 6B). These differences were most prominently seen in the anterior AF, where the collagen lamellae appear as a green/yellow color. These data indicate a difference in birefringence of collagen fibers in the H-AGE and L-AGE females.

### Dietary AGEs impaired collagen quality in female IVDs

**Fig. 7.**
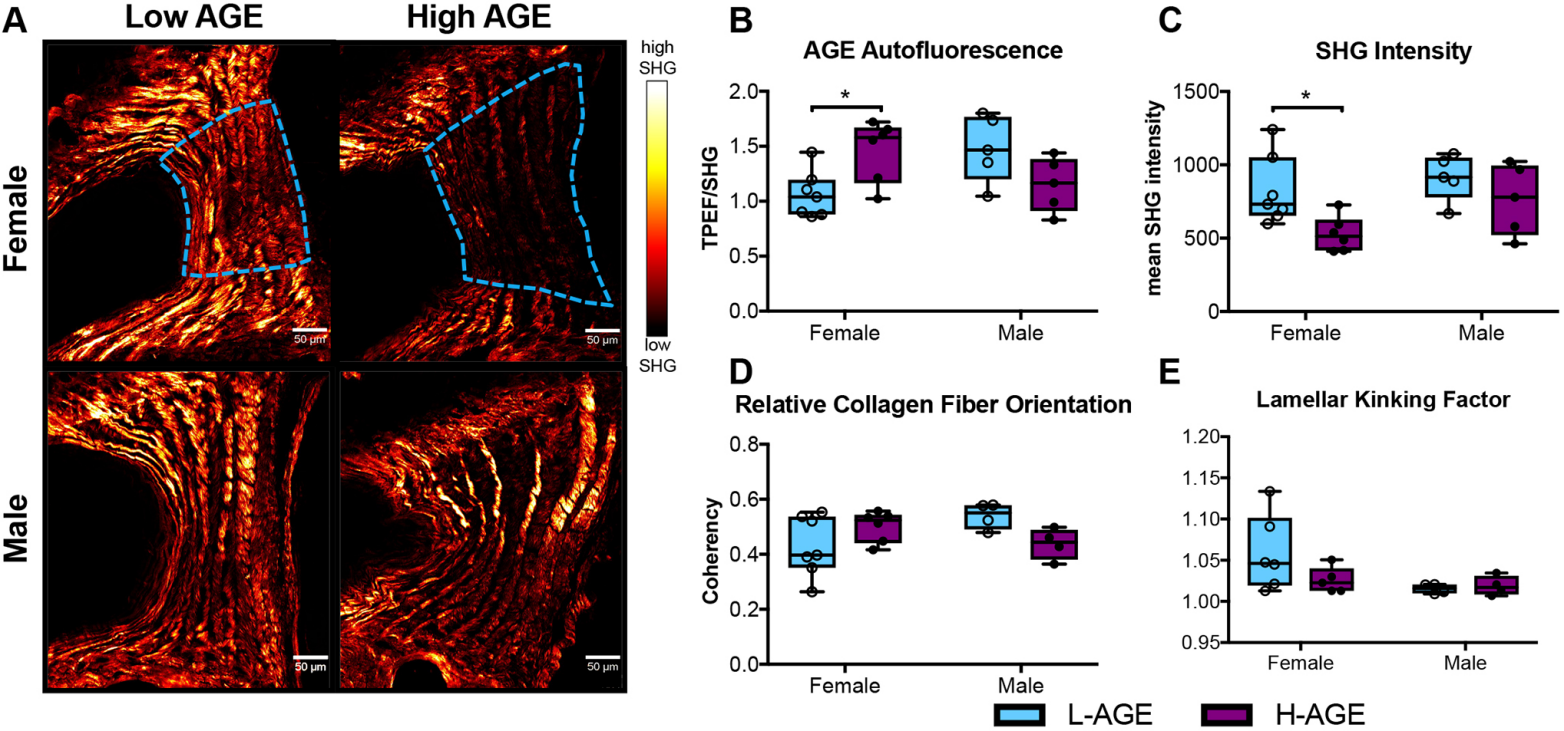
**SHG of collagen in annulus fibrosus (AF) tissue.** (A) Representative images with region of interest outlined in blue. (B) AGE autofluorescence increased and (C) SHG intensity was reduced in H-AGE female (‘F’) (*n*=6) compared to L-AGE F (*n*=7), with no differences in (D) relative collagen fiber orientation or (E) lamellar kinking of the lamellae. No differences were detected between males (L-AGE M and H-AGE M, *n*=5 per group). Data are presented as box plots from minimum to maximum±s.d. *P*-values are based on two-tailed unpaired Student's *t*-test with Bonferroni correction and significant if *P*≤0.05 (*).

**Fig. 8.**
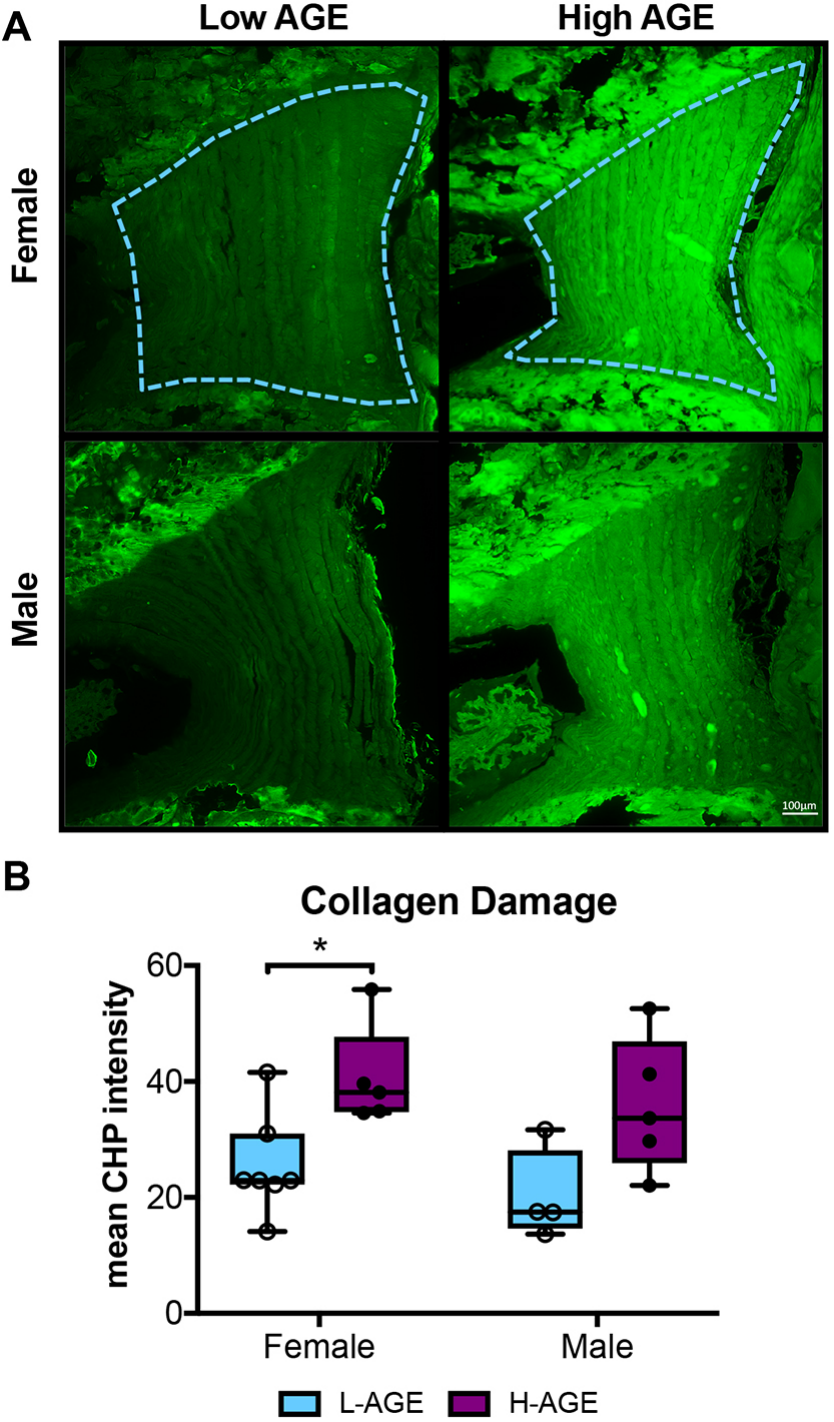
**Molecular assessment of collagen.** (A) CHP-stained fluorescent images of anterior AF exhibit increased CHP staining in H-AGE compared to L-AGE in females (‘F’) and males (‘M’). The region of interest is outlined in blue. (B) Quantification of CHP mean intensity shows significant collagen damage in H-AGE F (*n*=5) compared to L-AGE F (*n*=7). No differences were detected in between males (L-AGE M *n*=4, H-AGE M *n*=5). Data are presented as box plots from minimum to maximum±s.d. *P-*values are based on two-tailed unpaired Student's *t*-test with Bonferroni correction and significant if *P*≤0.05 (*).

To investigate collagen quality, we used two-photon imaging to quantify collagen second harmonic generation (SHG) intensity and a collagen hybridizing peptide (CHP), which reveals damaged collagen molecules. In female, but not in male, mice, SHG measured in the anterior AF appeared less bright in H-AGE compared to L-AGE photomicrographs (Fig. 7A). This was confirmed with quantitative measurements of the SHG intensity, which was significantly reduced in female H-AGE compared to L-AGE IVDs (*P*=0.037, Fig. 7C). Furthermore, in the same region, measurement of two-photon excitation fluorescence (TPEF)/SHG, or total AGE auto fluorescence (Marturano et al., 2014), was increased in female H-AGE compared to L-AGE IVDs (*P*=0.03, Fig. 7B). CHP staining of the same histological sections revealed significantly increased CHP (green fluorescence) staining in the AF of female H-AGE compared to L-AGE IVDs (*P*=0.03, Fig. 8), indicating greater collagen damage. Alignment of collagen fibers (measured through fiber coherency) or lamellae organization (measured through a kinking factor) appeared to not be affected by AGE accumulation. No differences for any parameter were observed in male mice. These data indicate that H-AGE diet led to AGE accumulation and collagen damage in the AF of female mice only.

## DISCUSSION

It is a priority to identify sources for structural disruption and matrix damage in IVD degeneration since these changes are known causes for painful conditions in the spine. AGE accumulation in tissues is a natural function of aging but is accelerated in conditions such as DM and can be increased by consumption of poor diets, by formation of endogenous AGEs in diets that are high in fructose (Cannizzaro et al., 2017) and/or by consuming exogenous AGEs that are formed in high-temperature processed diets (Tessier et al., 2016; Koschinsky et al., 1997). For this study, we processed standard chow with high-temperature treatment to increase exogenous AGEs. We demonstrated that, especially for females, consumption of this processed diet that is high in AGEs resulted in IVD AGE accumulation, which was associated with IVD functional changes, including increased compressive stiffness, increased torque range and torque failure properties. Significant alterations in collagen quality were observed and the significantly increased AGE crosslinking was most likely responsible for the observed biomechanical changes in these young animals. Increased collagen damage was also observed and will likely increase failure risk as IVDs age. These effects of H-AGE diet on IVDs were sex dependent, since only female mice were significantly affected and no significant effects were detected for males, although multiple parameters had means following similar trends as females. Moreover, the absence of hyperglycemia and obesity also suggests that the majority of AGEs measured in the IVDs and subsequent functional and structural changes in this study were a direct result of the exogenous AGEs.

Exogenous dietary AGEs have been shown to directly sequester in several tissues, including the liver, kidney (He et al., 1999) and IVD endplates (Illien-Jünger et al., 2015). We show for the first time that H-AGE consumption led to accumulation of the ingested AGEs within IVD tissues. In our study, AGE content in female H-AGE mice was increased about 150% in IVDs and 80% in circulation when compared to L-AGE mice. The differences in the amount of AGE accumulation is likely due to the slow collagen turnover in IVDs, which stands in contrast to the high turnover in the blood circulation. Our measured circulating AGEs are in line with clinical studies where circulating AGEs in type 2 DM patients were about 80% higher compared to non-DM patients (Kilhovd et al., 1999), while aging (>60-year-old women compared to <45-year-old women) was associated with 60% increase in circulating AGEs (Uribarri et al., 2007). This study thus further validates the significant contribution of dietary AGEs to total tissue AGEs and their ability to specifically accumulate in the IVD.

AGEs, including pentosidine, crosslink proteins to alter tissue structure and function, and can be a marker of protein aging and turnover (Sivan et al., 2006). The propensity to crosslink long-living proteins such as aggrecan and collagen has been shown to increase stiffness and brittleness in tissues such as articular cartilage (Verzijl et al., 2002), bone (Vashishth et al., 2001) and even the IVD (Wagner et al., 2006). AGE collagen crosslinks accumulate in IVDs with aging, being implicated with loss of IVD integrity and advancing the pathogenesis of the degenerative process (Pokharna and Phillips, 1998). Moreover, *in vitro* glycosylation of the AF specifically increases tissue stiffness and brittleness (Wagner et al., 2006). As shown in this study, AGEs can also accumulate within the IVD due to poor diet, leading to functional changes to the IVD.

The majority of biomechanical changes observed in this study are consistent with increased AF collagen crosslinking from AGE accumulation in the IVDs. The significantly greater values for torque range and torque to failure in females with H-AGE diet indicated that AF collagen fibers have increased stiffness. The greater TPEF/SHG ratio for this same group further suggests that this increased stiffness is due to AGE accumulation and crosslinking. The significantly increased compressive stiffness in the female H-AGE diet group is also likely due to crosslinking that increased AF collagen stiffness to resist radial bulging during compressive loading. In support of this concept, IVD motion segments exhibited increased compressive stiffness with increased collagen crosslinking from genipin (Barbir et al., 2010), although, with the IVD structural biomechanical testing of this study, we cannot reject the possibility that alterations in the nucleus pulposus or endplate can also play a role in this increased compressive stiffness.

It is noteworthy that we did not observe differences in tensile stiffness during axial tension-compression testing since AF collagen fibers also resist tension when tensed axially, and this is consistent with the ∼±30° AF fiber orientation and the finding that exogenous crosslinking caused a greater increase in circumferential tension than axial tension (Chuang et al., 2007). The lack of differences in IVD cross-sectional area between groups suggests that changes in material quality and not geometric differences are playing the largest role in contributing to the observed biomechanical behaviors. Increased collagen damage could act in opposition to the increased crosslinking to reduce sensitivity of some of these structural biomechanical properties to specific collagen changes. Collagen damage can be caused by increased crosslinking due to glycation (Tseng et al., 2010; Kim et al., 2000) and structural disorder of the collagen fibrillar array (Reiser et al., 2007). Glycation has been shown to disrupt collagen organization in multiple ways: AGEs can alter fibril organization through increased fibril packing and diameter (Bai et al., 1992; Hwang et al., 2011); AGE adducts can twist and distort the collagen fibrillar array (Kim et al., 2000); and AGEs can alter molecular packing by increasing the distances between collagen chains and molecules (Tanaka et al., 1988). While we did not directly measure the changes to collagen behavior, alterations in fibril packing can increase the susceptibility to enzymatic digestion (McBride et al., 1997) and collagen degradation, which in turn can contribute to the loss of collagen SHG intensity (Hwang et al., 2017).

CHP is a highly sensitive measure of collagen damage, which may not have accumulated sufficiently in these samples to result in biomechanical differences. The fiber-reinforced laminated composite structure of the AF has multiple levels of redundant fibers that are highly effective at maintaining function and resisting crack propagation (Iatridis and Ap Gwynn, 2004), so it is not surprising that damage was observed histologically but not biomechanically. Additionally, the spine undergoes various motions that combine complex loading conditions that accumulate with aging, yet even damaged collagen fibers would require high magnitudes of loading and/or older aged animals to significantly degrade biomechanical function to detectable levels. While our data suggest that biomechanical changes are partly driven by AGE crosslinking in the AF, we also observed that these pro-oxidative AGEs led to collagen damage, which is likely to increase risk of IVD injury under higher loading conditions or in older mice. It has been proposed that AGE crosslinking can lead to mechanically induced breakdown of the collagen triple-helical structure by causing forces, transmitted through the intermolecular AGE crosslinks, to accelerate enzyme degradation (Bourne et al., 2014). However, AGEs can also induce enzymatic activity and matrix catabolism, for example by binding to the receptor for AGEs (RAGE) (Ott et al., 2014). Some have suggested an important role for the AGE/RAGE pathway in DM and AGE-induced IVD degeneration (Fields et al., 2015; Illien-Jünger et al., 2016); however, future studies will investigate the role of this interaction further, especially as it pertains to dietary AGEs. Together with the literature, our assessment of SHG intensity and CHP binding suggests that dietary AGEs lead to increased glycation within the AF collagen matrix, inducing collagen fibrillar disorder and molecular damage.

This study comes with some limitations. First, we did not identify any qualitative histological differences in the nucleus pulposus or endplate for any groups. While AB staining is sensitive to glycosaminoglycan content, this measurement has limited capacity to detect crosslinking and hydration changes so that further characterization of nucleus pulposus changes would be required to fully reject the possibility that alterations in the nucleus pulposus or endplate may also play a role in this increased compressive stiffness of the complex IVD structure. Second, there were large differences in chow consistency so that reliable comparison of chow consumption between diets was not possible, and it is not clear whether the consistency affected chow consumption. Our data indicate that mice on H-AGE diet consumed 1 g/day (1/3 of the diet) less compared to L-AGE; however, body weights were in the normal range whereas we would have expected an increase, making comparison between diets questionable. Nevertheless, within diets, chow consumption did not differ between sexes, suggesting that the sex-dependent effects of AGE intake might be due to differences in the metabolism of female and male mice. A third limitation is that we did not control for the estrus cycle, which may have contributed to a greater variance within the female data, although this did not seem to be important in our measurements since many significant differences were identified for female but not male IVDs. Nevertheless, causes for the sex-dependent effects of dietary AGEs warrant future investigation, especially because estrogen is known to regulate cell metabolism (Mauvais-Jarvis et al., 2013) and to influence RAGE (Tanaka et al., 2000). In addition, we previously have shown that dietary AGEs affect bone mechanical properties mostly in young female mice (Illien-Jünger et al., 2018), further emphasizing the importance of assessing male and females separately. Lastly, the variations in vertebrae and IVDs also highlight the importance of evaluations of all spinal structures, which interact and may influence each other.

In conclusion, H-AGE diets can be a source for IVD crosslinking and collagen damage, which are known to be important in IVD degeneration and can result in changes to functional IVD biomechanical behaviors. These effects of dietary AGE on the spine were significant for females but not males, highlighting the importance of sex-dependent effects on spinal tissues. Results suggest that dietary and other interventions that modify AGE levels warrant further investigation for promoting spinal health. Due to the increased risk for AGE accumulation with hyperglycemia in DM, we further conclude that targeting AGEs may be particularly important in promoting spinal health in DM patients.

## MATERIALS AND METHODS

### Mouse model

After weaning, 21 female and 23 male C57BL/6J mice were each assigned to two isocaloric diet groups, receiving either a low AGE chow (L-AGE; containing 7.6 µg/mg AGE; Test Diet Low AGE 5053; WF Fisher & Son CO, NJ, USA) or high AGE chow, generated via high-temperature heating (H-AGE; 40.9 µg/mg; NIH-31 open formula chow autoclaved for 30 min at 120°C, Table S1), which has been shown to contain high amounts of carboxymethyl lysine, methylglyoxal-H1 and other lipid-derived AGEs (Cai et al., 2008). H-AGE diet was ∼80% higher in AGEs and resembled a human western diet in which chicken might be fried (e.g. crispy McDonalds chicken 7722 AGEs kU/100 g) or microwaved (skinless chicken breast 1524 AGEs kU/100 g) (Uribarri et al., 2010). Food consumption was recorded once a week for the last month of the study period and daily food consumption per mouse was averaged between the number of mice per cage.

To exclude the effect of maternal AGEs on the experimental mice (Mericq et al., 2010; Peppa et al., 2003), offspring of mice that were bred on the respective diets for at least three generations were used for this study. Mice were group housed and had *ad libitum* access to food and water. Mice were euthanized at 6 months of age by cardiac puncture. Prior to sacrifice, body weights and fasting blood glucose measurements (16-h fast) were acquired to establish body habitus and diabetic status. All experiments were performed in accordance with Mount Sinai Institutional Animal Care and Use Committee.

### Tissue harvest

Following sacrifice, caudal (C) IVDs from C5 through C10 were carefully dissected from each tail and stored at −80°C until use for western blot analysis. For biomechanical testing, C4-C5 bone-IVD-bone motion segments were dissected from mouse tails. Motion segments were prepared by making a parallel cut (transverse to the long axis of the spine) through the C vertebrae and included the C4-C5 IVD. The facet joints and all surrounding soft tissue were removed. Motion segments were wrapped in phosphate buffer saline (PBS)-soaked paper towels and stored at −80°C until the day of testing. Lumbar (L) L3-L4 and L4-L5 segments were dissected and fixed in 10% neutral buffered formalin prior to resin embedding for histological analysis (Table S2).

### Fasting blood glucose and serum AGE measurement

At sacrifice, blood was collected for analysis of fasting glucose (Aimstrip Plus Glucose Meter; Germaine Laboratories, San Antonio, TX, USA) and serum AGE quantification. Total serum AGEs were measured with a competitive ELISA (developed in our laboratory) using an anti-AGE antibody (Abcam, Cambridge, MA, USA; ab23722). For this study, 1 unit (U) corresponds to 3 mg AGE-BSA.

### Protein measurements for AGE quantification

For protein extraction, IVDs were placed in lysis buffer (T-PER tissue protein extraction reagent, Thermo Scientific, Waltham, MA, USA)+protease inhibitor (Protease inhibitor cocktail, Bimake, Houston, TX, USA) and sonicated. Following centrifugation (10,000 ***g***), supernatants were collected and proteins were separated by sodium dodecyl sulphate polyacrylamide gel electrophoresis (SDS-PAGE; Bio-Rad Laboratories, Hercules, CA, USA). A total of 20 μg protein samples were loaded on gradient gels (4-20%; Bio-Rad Laboratories). After electrophoresis, proteins were transferred on nitrocellulose blotting membranes (Protran BA Cellulosenitrat; Schleicher & Schuell, Dassel, Germany). Following the protein transfer, membranes were incubated for 30 min in blocking buffer (5% milk powder in PBS+0.2% Tween^®^ 20) and then incubated with the primary antibodies, AGE (1:3000; ab23722; Abcam, Cambridge, MA, USA) and GAPDH (internal control; 1:20,000; ab181602; Abcam) overnight at 4°C. AGE-BSA protein (ab51995; Abcam) was used as a positive control for AGE detection. The next day, the membranes were washed in washing buffer (PBS+0.2% Tween^®^ 20) and incubated with an Eu-labeled secondary antibody (Molecular Devices, San Jose, CA, USA) for 1 h at room temperature. After washing, the membranes were dried and scanned using the ScanLater (Molecular Devices) Western Blot cartridge in a SpectraMax i3 system (Molecular Devices). Membranes were analyzed by ScanLater software.

### Biomechanical testing

All samples underwent only one freeze-thaw cycle since this can influence IVD mechanical properties (Azarnoosh et al., 2017). Prior to mechanical testing, motion segments were thawed and hydrated in cold 1× PBS for 5-10 min, and IVD diameters were measured using a caliper. Caudal motion segments were used for biomechanical testing due to their cylindrical geometry and relatively large IVD and vertebral heights that make gripping and testing simpler than using lumbar motion segments for these small mouse samples. Previous studies have compared torsional and compression mechanical behavior of rat lumbar and caudal discs and shown that caudal IVDs have a somewhat larger neutral zone and lower stiffness than lumbar spinal regions (Espinoza Orías et al., 2009; Sarver and Elliott, 2005).

Axial compression-tension and torsional testing was performed on the ElectroForce 3200 and the AR2000x Rheometer (TA Instruments, New Castle, DE, USA), respectively. Axial and torsional loading were applied separately since these simplified loading modes allow us to determine and interpret biomechanical properties. Torsional properties are largely driven by the AF collagen integrity and quality since the shear stresses developed are largest in the outer AF region and must be resisted by AF collagen fibers (Iatridis et al., 2013; Showalter et al., 2012). Axial compression-tension testing involves a more complex stress state in the AF, nucleus pulposus and endplate due to nucleus pulposus pressurization, AF compression, radial bulging and direct AF tension. While axial compression-tension properties are known to be sensitive to alterations in nucleus pulposus pressurization and hydration, they can also be influenced by AF and endplate changes (Boxberger et al., 2006; Iatridis et al., 2013; Showalter et al., 2012). For axial testing, each motion segment was placed in a 1× PBS bath at room temperature between custom-designed fixtures and microvises. The test consisted of 20 cycles of tension and compression with peak loads of 0.5 N applied at a 0.1 mm/s displacement rate (Elliott and Sarver, 2004). To recover IVD height after axial testing, motion segments were placed into a 1× PBS bath for 5 min prior to torsional testing. Following an axial preload for 5 min at 0.1 MPa axial stress (Showalter et al., 2012), torsional testing was performed and consisted of 20 cycles of rotation to ±10° at a frequency of 1 Hz followed by a 1°/s continuous rotation to failure (Michalek et al., 2010). Sample sizes were initially balanced yet final numbers vary between groups because some motion segments failed during tension-compression or torsion testing and thus were excluded from analysis. A custom-written MATLAB code was used for calculating compressive stiffness, tensile stiffness, axial range of motion, torsional stiffness and torque range using the second to last cycle of every test. Stiffness measurements were defined as the slope of 20% of the top and bottom (linear region) of the force-displacement curves (Fig. 2). Torsional stiffness is reported as the average of clockwise and counter-clockwise measurements. Ultimate failure torque and degree were calculated manually from the force-displacement curves and the work done to failure was calculated as the area under this curve.

### Histological assessment

#### IVD morphology

Fixed, calcified lumbar segments were embedded in poly(methyl methacrylate) (PMMA) and 5 μm sagittal sections were cut. Mid-sagittal sections were stained with PR/AB to assess collagen and proteoglycan content, respectively. PR/AB-stained sections were then imaged under DIC using the Axio Imager Z1 (Zeiss, Oberkochen, Germany) to assess overall IVD morphology and composition. The same samples were then imaged under polarized light using the LEICA DM6 B (Leica Microsystems, IL, USA) for qualitative assessment of AF collagen organization and arrangement. For all histological analysis, while sample sizes were initially balanced across groups, final numbers vary in each group because only mid-sagittal sections were used for comparison and any non-sagittal sections were excluded from analysis; any outliers due to technical errors with staining were also excluded.

#### Molecular assessment of collagen

PMMA-embedded mid-sagittal lumbar sections were stained using collagen hybridizing peptide-biotin conjugate (B-CHP; BIO300, 3Helix Inc; Salt Lake City, UT, USA) to detect the presence of collagen damage as described previously (Hwang et al., 2017; Zitnay et al., 2017). This procedure includes the creation of a 2 µM CHP solution, heating of the solution at 80°C for 5 min to monomerize the peptides, quenching in an ice bath for 30 s, and application of the solution to the tissue. The tissue was incubated overnight, and then positive binding was detected using GFP-labeled streptavidin (Dylight 488 Strepavidin; Vector Laboratories Inc., Burlingame, CA, USA).

### SHG imaging

Unstained resin-embedded sections were imaged with an Olympus FV1000 MPE laser-scanning microscope (Olympus Corporation, Tokyo, Japan) at the Icahn School of Medicine at Mount Sinai Microscopy Core. Two-photon excitation was done with the tunable Coherent Chameleon Vision II laser. Backward signal propagation was collected using the dedicated Olympus WLPLN water immersive 25× objective with a numerical aperture of 1.05. A stepwise approach was used to capture the SHG signal of fibrillary collagen and the TPEF signal of total AGE autofluorescence (Tang et al., 2007; Vashishth, 2009) in the anterior AF of each sample. Excitation for backward SHG (B-SHG) was performed at 910 nm and recorded by a photomultiplier tube (PMT) at 440±10 nm. The laser was then tuned to 740 nm and the TPEF was recorded with the same detector at 440±10 mm. All parameters (i.e. laser intensity, gain, voltage, dwell time, aspect ratio) were held constant for the SHG and the TPEF imaging to allow comparison of image intensity. Optical slices were taken through the entirety of every section at a step size of 1.5 µm and a maximum intensity *z*-projection was performed for both SHG and TPEF. The B-SHG and TPEF intensities were assessed by measuring the average pixel intensity/area of the anterior AF using NIH-ImageJ (http://rsbweb.nih.gov/ij/).

### Fiber orientation analysis

Collagen orientation was quantified from the B-SHG images using OrientationJ, a series of plugins for ImageJ (http://bigwww.epfl.ch/demo/orientation/), as described by Rezakhaniha et al. (2012). Briefly, the color balance was manually adjusted to maximize the observer's ability to distinguish AF fibers. Next, a Laplacian of Gaussian prefilter (σ=1.0) was applied to decrease noise and enhance the edge detection. Fiber orientation was then calculated based on the gradient of intensity changes so that it was independent of image properties. The fiber coherency is a relative measure of fiber orientation that indicates whether the local fibers within each lamellae are orientated in the same direction, where a value of 1 indicates that all fibers are in the same orientation and a value of 0 indicates fibers that are isotropic. Lamellae were outlined as a region of interest and selected based on the following criteria: adjacent lamellae have alternating dominate directions; each lamella spans the IVD height, and the inter-lamellar boundaries were easily identified from abrupt changes in a dominant fiber direction. Lamellar kinking factor was calculated as the ratio of the lamellar length to shortest linear distance to the ends of the lamella, where a value of 1 indicates that the lamella is perfectly straight. For each IVD, coherency and kinking factor were calculated from the average of the same four adjacent lamellae from the anterior AF.

### Statistics

All data are presented as box plots with minimum, first quartile, median, third quartile and maximum values, with error bars representing the standard deviation. Two-tailed unpaired Student's *t*-test with Bonferroni correction was applied between L-AGE and H-AGE females and between L-AGE and H-AGE males. All data were considered significant if *P*≤0.05 and a trend if *P*≤0.10.

## Supplementary Material

Supplementary information
