# Supplementary material for: Dietary advanced glycation end-product consumption leads to mechanical stiffening of murine intervertebral discs
Source: Dis Model Mech. 2018 Dec 18;11(12):dmm036012. doi: 10.1242/dmm.036012 (PMC6307905; doi:10.1242/dmm.036012)
Supplement: Supplementary information [file dmm-11-036012-s1.pdf]

Table S1. Experimental Groups

C57BL/6J mice	Low AGE Diet (L-AGE) 7.6 µg/mg AGE	High AGE Diet (H-AGE) 40.9 µg/mg AGE
Female	n=10	n=11
Male	n=13	n=10

Table S2. Primary Study Measurements

TISSUE	TEST	MEASUREMENT
Blood	Serum ELISA	Blood glucose<br>AGE measurement
Caudal IVD	Western Blot	AGE measurement
Caudal IVD C4-C5	Mechanical Testing	Axial, Torsional and Failure tests
Lumbar IVD L4-5	Histology<br>(Picrosirius/Alcian blue)	DIC, polarized light & SHG imaging<br>collagen denaturation
